# Development and validation of a proposed rule for estimating central venous pressure from inferior vena cava dynamics: a clinical prediction model study

**DOI:** 10.1186/s44158-026-00438-z

**Published:** 2026-07-29

**Authors:** Sameh M. Hakim, Nermeen S. Ahmed, Sahar M. Talaat, Sana F. Wasfy, Adham M. Haggag

**Affiliations:** Department of Anesthesiology, Intensive Care, and Pain Management, Ain Shams University Faculty of Medicine, 15 Gamal Noah Street, Heliopolis, Almaza, Cairo, 11341 Egypt

**Keywords:** Central venous pressure, Prediction rule, Clinical, Ultrasonography, Vena cava, Inferior

## Abstract

**Background:**

This study aimed to derive and validate a prediction rule for estimating the central venous pressure (CVP) from the inferior vena cava collapsibility index (IVCCI) in adults.

**Methods:**

Five hundred forty paired CVP-IVCCI measures were obtained from 180 adults. The CVP was measured via a central venous catheter connected to a pressure transducer, and the IVCCI was estimated by abdominal ultrasound. The dataset was randomly split at the patient level into training (121 patients, 363 observations) and validation (59 patients, 177 observations) samples. A generalized estimating equation for repeated measures was used to fit a prediction rule from the training sample, which was validated on the validation subset.

**Results:**

The model showed promising performance with a mean absolute error of 2.13 mmHg and 75.7% of predictions falling within ± 3 mmHg of the true values. The limits of agreement ranged from –6.0 to + 4.3 mmHg, 85.9% of predictions fell within a prespecified maximum accepted difference of ± 4 mmHg, and the direction of change was correctly predicted in 65.3% of instances. Calibration analysis showed the model tended to systematically underestimate high values and vice versa, but recalibration by applying a shrinkage factor worsened the model’s performance.

**Conclusions:**

The suggested rule exhibits promising performance for most instances, rendering it useful for non-invasive trend monitoring and triaging purposes. However, single-point estimates carry considerable uncertainty that precludes their use for definitive, high-stakes clinical decisions. Validation on larger cohorts and recalibration utilizing more elaborate modeling methods are recommended before it is adopted for regular use.

**Trial registration:**

The trial was prospectively registered at the clinicaltrials.gov registry (https://register.clinicaltrials.gov/, registration number: NCT06166875, registration date: 4 December 2023).

**Supplementary Information:**

The online version contains supplementary material available at 10.1186/s44158-026-00438-z.

## Introduction

Central venous pressure (CVP) is a fundamental parameter for assessing right heart function and intravascular volume status. Regularly measured invasively via a vascular catheter inserted into a central vein, the CVP serves as a critical guide for fluid management in various clinical settings, including the operating room, emergency department, and intensive care unit [1]. Nonetheless, central venous catheterization is an invasive procedure that introduces risks such as infection, thrombosis, and pneumothorax, and requires technical expertise and monitoring infrastructure that may not always be available [2].

With the current shift in clinical practice toward less invasive monitoring techniques, the inferior vena cava collapsibility (IVCCI), which quantifies the respiratory variation in inferior vena cava (IVC) diameter, has emerged as a potential non-invasive surrogate for direct measurement of the CVP through intravascular catheters [3], and as a predictor of volume responsiveness, particularly in spontaneously breathing patients [4].

The physiological basis linking the IVC dynamics and right atrial pressure (RAP) stems from the direct communication between these structures, rendering the phasic changes in IVC diameter with respiration susceptible to changes in the intrathoracic pressure and volume status [5], which is less consistent in mechanically ventilated patients, owing to the reversal of intrathoracic pressure changes associated with positive pressure ventilation [6].

Several studies have explored the correlation between the IVCCI and invasively measured CVP [7–17] or determined a cutoff for the IVCCI to identify patients with high or low CVP values [8, 10, 13–17]. However, fewer studies attempted to provide a prediction rule to estimate the CVP from the IVC respiratory dynamics as measured with ultrasound. To our knowledge, one such equation was modeled to calculate the CVP in mechanically ventilated patients [11], and another rule estimated the RAP in adults with congenital heart disease (CHD) [12], neither of which has been previously validated.

In this regard, the present study fills a literature gap as it aimed to fit and validate a mathematical rule for the non-invasive estimation of CVP in adults admitted to the intensive care unit (ICU), who by far represent the largest patient population in whom the filling pressure is required to be measured [18].

## Materials and methods

This cross-sectional, single-center, clinical prediction model study was conducted during the period from December 2023 to April 2025 at a university hospital, in compliance with the Helsinki Declaration as revised in 2024, and the *T*ransparent *R*eporting of a multivariable prediction model for *I*ndividual *P*rognosis *O*r *D*iagnosis (TRIPOD) Statement [19]. The study was approved by the institutional review board and registered at the clinicaltrials.gov registry. Informed consent was obtained from all patients or their surrogates.

### Eligibility criteria

Spontaneously breathing adult patients (≥ 18 years of age) of either sex, who were admitted to the ICU and required a central venous catheter (CVC) for their management, were eligible. Patients admitted to the ICU after surgery with a CVC inserted in the operating theater were also included, as were those who had been weaned from mechanical ventilation (MV), non-invasive ventilation (NIV), or continuous positive airway pressure (CPAP) for > 24 h. Exclusion criteria were body mass index (BMI) ≥ 35 kg/m^2^, pregnancy, or increased intra-abdominal pressure (IAP) (≥ 12 mmHg) of any cause. IAP was measured invasively via an indwelling urinary catheter, if already inserted, which was connected to a standard pressure transducer system. Otherwise, IAP was assessed non-invasively with abdominal ultrasound using the method described by See et al*.* [20]. Other exclusion criteria were the inability to perform IVC ultrasound scans because of surgical incisions or drains, or the need for prolonged (≥ 21 days) ventilatory support (MV or NIV/CPAP).

### Technique of central venous cannulation

All patients had a CVC inserted via the right internal jugular vein (IJV) using Selinger’s technique and utilizing a middle approach under ultrasound guidance. As per our institution’s protocol, CVCs are advanced empirically to a depth equal to the distance from the insertion point to the right sternoclavicular joint plus the distance from the latter to the lower border of the right 2nd rib, aiming to place the CVC tip at the mid-superior vena cava. After insertion, a plain posteroanterior chest x-ray was obtained in the 30-degree head-up position to verify that the CVC tip placement was just above the carina, and the CVC depth was adjusted accordingly, if needed.

### Framework for acquiring the paired CVP and IVCCI measurements

Three paired CVP-IVCCI measurements were obtained from each patient at three prespecified time points, 07:00 h, 15:00 h, and 23:00 h. The rationale for this was to: (1) improve precision and statistical power by increasing the effective sample size while accounting for within-subject correlation, (2) capture temporal hemodynamic variability and assess the consistency of the IVCCI-CVP relationship across different time points, and (3) simulate real-world clinical practice. The order of measurements was standardized; the CVP reading was obtained first, followed by the IVCCI ultrasound measurements within a maximum interval of ≤ 5 min. This sequence was chosen because CVP measurement provides an immediate readout, while IVCCI acquisition requires additional time for ultrasound probe placement and image optimization. To minimize hemodynamic variability during the paired acquisitions, no other clinical interventions, such as fluid boluses, vasopressor adjustments, or diuretic administration, were carried out during the measurement session or within 15 min preceding it, and the steadiness of respiratory pattern was ensured throughout the acquisition session. Take out.

### Technique of CVP measurement

Three CVP measurements were obtained from each patient at 8-h intervals starting from 7 AM. The measurements were acquired using a pressure transducer connected to the CVC, with the patient placed in the supine position and the phlebostatic axis (zero point) for the transducer located at the intersection of the midaxillary line with the 4th intercostal space [21]. For patients who could not tolerate the decubitus position, they were placed in a 30 to 45-degree head-up (semi-sitting) position, and the pressure transducer was repositioned, level with the standard phlebostatic axis [22]. Three consecutive measurements were taken by the same observer at each time point and were then averaged to obtain the CVP. Two intensivists not participating in the study took all CVP measurements.

### Technique of IVCCI measurement

The IVCCI measurements were obtained by one of two intensivists (NSA and AMH) who acquired formal training in abdominal sonography and were not involved in measuring the CVP. Unless it was not tolerated, all the examinations were conducted in the supine position. Otherwise, a 30 to 45-degree head-up position was employed [23].

**Fig. 1:**
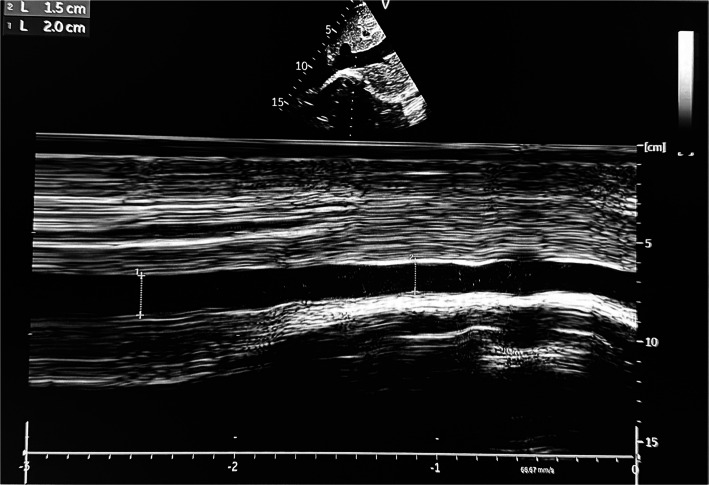
M-mode ultrasound scan of the inferior vena cava (IVC) in the long-axis view acquired in a spontaneously breathing patient utilizing a subxiphoid approach. The transducer was placed inferior to the xiphoid process and angled cephalad to visualize the IVC in its long axis at its confluence with the hepatic veins. The IVC measures were acquired approximately 2 cm caudad (downstream) to the hepatic vein-IVC junction. The maximum anterior–posterior IVC diameter (IVCd_max_) was measured at the end of expiration from the hyperechoic inner layer of the near wall to the hyperechoic inner layer of the far wall. The minimum anterior–posterior IVC diameter (IVCd_min_) was measured at the end of inspiration using the same technique. On the scan, the maximum and minimum IVC diameters are illustrated by *Line 1* and *Line 2*, respectively. The IVC collapsibility index (IVCCI) was calculated using the following formula: $\mathrm{IVCCI} (\%) = \frac{{\mathrm{IVCd}}_{max}-{\mathrm{IVCd}}_{min}}{{\mathrm{IVCd}}_{\mathrm{max}}}\times 100$. *IVC*: inferior vena cava, *IVCCI*: inferior vena cava collapsibility index, *IVCd*_*max*_: maximum inferior vena cava diameter, *IVCd*_*min*_: minimum inferior vena cava diameter

A portable ultrasound system (M-Turbo, Fujifilm Sonosite, Inc., Bothell, WA, USA) equipped with a phased-array transducer (P21x, 1–5 MHz) was used to visualize the IVC in the long-axis subxiphoid view, using M-mode ultrasound. The transducer was placed inferior to the xiphoid process and angled cephalad, using the left lobe of the liver as a sonic conduit to visualize the IVC in its long axis at its confluence with the hepatic veins. The IVC measures were acquired approximately 2 cm caudad (downstream) to the hepatic vein-IVC junction. The maximum anterior–posterior IVC diameter (IVCd_max_) was measured at the end of expiration using the leading-edge technique, *i.e.*, from the hyperechoic inner layer of the near wall to the hyperechoic inner layer of the far wall. The minimum anterior–posterior IVC diameter (IVCd_min_) was measured in the same anatomical location at the end of inspiration. The respiratory phases were distinguished by direct visual inspection of the chest and abdominal wall movements and were simultaneously confirmed by real-time observation of the IVC diameter changes on the M-mode ultrasound display, where inspiration and expiration in a spontaneously breathing patient were characterized as collapse or expansion of the IVC, respectively. To minimize the effect of ordinary breath-to-breath variations, three consecutive respiratory cycles were recorded and averaged for each time point. The IVCCI was calculated using the following standard formula: $\mathrm{IVCCI} (\%) = \frac{{\mathrm{IVCD}}_{\max} - {\mathrm{IVCd}}_{\min}}{{\mathrm{IVCd}}_{\max}}\times 100$, where IVCCI = inferior vena cava collapsibility index, IVCd_max_ = maximum IVC diameter, and IVCd_min_ = minimum IVC diameter. Figure 1 shows an ultrasound scan illustrating the acquisition of the IVC measures.

## Statistical methods

### Sample size calculations

The target sample size for this study was 180 patients, each contributing three repeated CVP and IVCCI measurements (total = 540 paired measurements). This corresponded to an effective sample size of 100 subjects after accounting for within-subject correlation, which is the recommended minimum number for the development of prediction models [24]. The effective sample size was calculated using the methods described by Liu & Colditz [25]. Aiming to divide the whole dataset into a training (70%) and validation (30%) subset [26], the validation sample was estimated to include approximately 162 observations, which exceeded the recommended minimum of 100 observations for reliable assessment of model performance in validation studies [27].

### Statistical software

Statistical analysis was performed using the IBM SPSS Statistics for Windows, Version 27.0 (IBM Corp., Armonk, NY, USA). Python version 3.10.12 with the *scikit-learn* (v. 1.2.2), *numpy* (v. 1.23.5), *pandas* (v. 1.5.3), *matplotlib* (v. 3.7.1), and *scipy* (v. 1.10.1) packages were used for stratified cross-validated receiver-operating characteristic (ROC) analysis. The following Python packages were used for repeated-measures Bland–Altman analysis with ordinary least squares regression: *pandas* (v. 1.5.3), *numpy* (v. 1.23.5), matplotlib (v. 3.7.1), *scipy* (v. 1.10.1), and *statsmodels* (v. 0.13.0). Calibration plots and statistical analyses were generated using Python (v. 3.10.12) with the following core packages: *matplotlib* (v. 3.7.1), *statsmodels* (v. 0.13.0), and *scikit-learn* (v. 1.2.2). A two-sided *P* value < 0.05 was considered statistically significant for all analyses.

### Data presentation and inferential statistics

Normally distributed variables were presented as mean ± standard deviation (SD) and categorical variables as counts and percentages. Test–retest and inter-observer reliability were assessed using the intraclass correlation coefficient (ICC) with 95% confidence intervals (CI). Correlations were examined using Pearson’s product-moment correlation. The diagnostic performance of the IVCCI for predicting low (< 5 mmHg) or high CVP (> 10 mmHg) was evaluated using a 10-fold stratified cross-validated ROC analysis.

### Dataset partitioning

To develop and validate the prediction model, the dataset of 540 paired CVP-IVCCI observations from 180 patients was partitioned into training and validation subsets, in a ratio of approximately 7:3, using independent Bernoulli sampling [25]. The split was carried out at a patient level, rather than an observation level, to avoid data leakage and ensure independence of the subsets, *i.e.*, to guarantee that all three observations from any single patient were assigned to one and the same of the two subsets. First, the dataset was aggregated at the patient level to create a single record per patient, ensuring that each patient contributed one unique identifier for the randomization process. A random number between 0 and 1 was then generated for each patient using a uniform distribution with a fixed random seed (12345) to ensure reproducibility. Subsequently, this random number was merged back into the original observation-level dataset, attaching the same random number to all three observations of each patient. Patients were then sorted by their random number and assigned to the training set if their random number was less than.7, and to the validation set if it was.7 or greater. This process yielded a training dataset comprising 363 observations from 121 (67.2%) patients and a validation dataset comprising 177 observations from 59 (32.8%) patients. The prediction model was fitted on the training cohort and was then independently validated and calibrated on the validation cohort [28].

### Modeling the CVP as a function of the IVCCI

A Generalized Linear Model utilizing the Generalized Estimating Equations (GEE) function was employed to model the CVP as a function of the IVCCI to account for the within-subject correlations [29]. To address the skewness and zero values in the CVP data, a Tweedie distribution [30] with a log link function [31] was specified. An exchangeable working correlation structure was selected, assuming equal correlation among the repeated measurements from each patient, which is valid for physiological parameters such as the CVP and IVCCI [29]. The Huber-White robust sandwich variance estimator was used to ensure valid standard errors, even if the correlation structure was misspecified [32, 33].

### Development of the training model from the training dataset

We explored the goodness of fit of alternative candidate models that included the intercept and IVCCI as a basic covariate, in addition to one or more of the following fixed effects: indication for ICU admission, time of measurement, and an interaction term between the IVCCI and indication for ICU admission. We selected a parsimonious model that included only the intercept and IVCCI to fit the training rule, as this was associated with the best goodness of fit, evidenced by the lowest Quasi-likelihood under Independence Model Criterion (QIC) and Corrected Quasi-likelihood under Independence Model Criterion (QICC) values [34]. The assumptions of the GEE were tested by examining the following metrics:

- Linearity and independence of the residuals: This was assessed by examining a scatter plot of the raw residuals versus predicted CVP values and the associated correlation coefficient (Pearson’s *r*).
- Normality of the residuals: This was assessed by examining the frequency histogram of the raw residuals and the associated skewness coefficient.

### Independent validation of the training model on the validation dataset

To independently evaluate the training model’s performance, we applied the prediction rule to the validation dataset and calculated the predicted CVP on the original scale as the exponentiated value of the log-linear link function of the model and calculated the following clinical performance metrics:

- Mean absolute error (MAE): This was calculated as the average of the absolute differences between the predicted (fitted) and observed (actual) CVP values.
- Root mean square error (RMSE): This was calculated as the square root of the mean of the squared errors.
- Percentage of predictions within clinically acceptable error margins: The proportion of predictions falling within ± 1 mmHg, ± 2 mmHg, ± 3 mmHg, and ± 4 mmHg of the observed CVP values was calculated.
- Correlation between the predicted and observed CVP values: This was evaluated using Pearson’s *r*.
- Agreement between the predicted and observed CVP values: This was assessed using a repeated-measures Bland–Altman analysis to account for the within-patient correlation arising from the three repeated measurements per patient [35]. To account for the within-patient correlation arising from repeated measurements, a mixed-effects model with a patient-specific random intercept was fitted to the differences (Predicted − Observed) to estimate the mean bias and the total variance [36]. The 95% limits of agreement (LoA) were calculated as bias ± 1.96 × SD. To obtain 95% CI for the bias and LoA that appropriately account for the clustered data structure, a clustered bootstrap with 5,000 resamples was performed, resampling patients (clusters) with replacement [37]. Proportional bias was assessed by regressing the differences on the mean of the two measurements; a slope significantly different from zero indicates proportional bias [38]. A maximum accepted difference (MAD) of ± 4 mmHg between predicted and observed CVP values was pragmatically prespecified as a clinically important difference.
- Agreement between changes in predicted CVP (Δ Predicted CVP) and changes in observed CVP (Δ Observed CVP) across consecutive time points: To assess the model’s utility for trend monitoring, we evaluated the agreement between Δ Predicted CVP and Δ Observed CVP across consecutive time points (Time 1 toTime 2 and Time 2 to Time 3) using a repeated-measures Bland–Altman analysis, following the same methodology described for the absolute CVP measurements [35–38].

### Calibration assessment

As an additional step, we applied the final model to the entire validation cohort (*n*=177 paired observations) to examine the potential for adjustment of the model’s estimations to improve its performance and enhance its clinical utility. This was carried out using three complementary approaches: (1) calibration-in-the-large (CITL), defined as the mean difference between observed and predicted CVP values, with a value of 0 indicating perfect overall calibration, (2) linear regression of observed on predicted CVP values to estimate the calibration slope (ideal = 1.0), calibration intercept (ideal = 0), and the coefficient of determination (*R*^*2*^) as a measure of the variance in the observed CVP that was accounted for by the predicted CVP, and (3) a calibration plot depicting observed CVP against predicted CVP, overlaid with a Local Regression Smoothing (LOESS) curve, together with an ordinary linear regression line and the ideal 45° line of perfect agreement [39]. These analyses were intended to diagnose potential miscalibration and guide future recalibration efforts, rather than to modify the original model’s parameters.

## Results

**Fig. 2:**
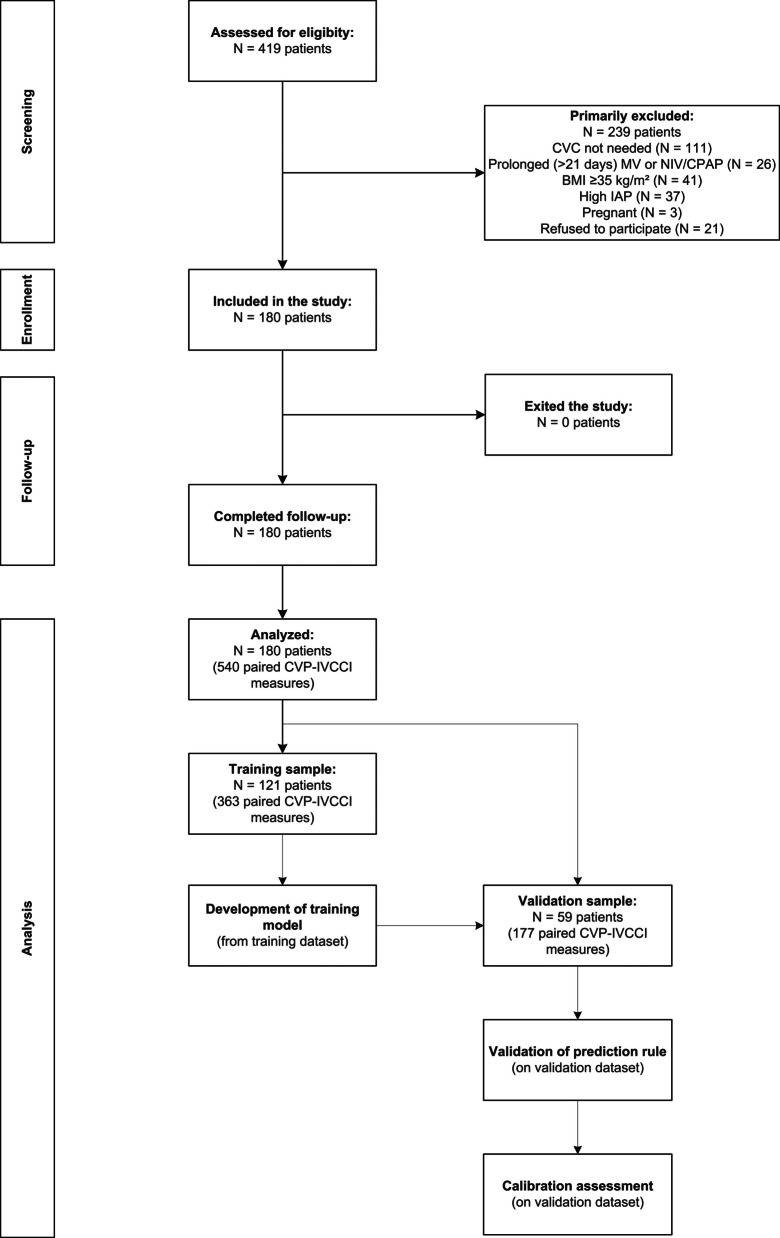
*T*ransparent *R*eporting of a multivariable prediction model for *I*ndividual *P*rognosis *O*r *D*iagnosis (TRIPOD) [19] flow chart. *BMI*: body mass index, *CVP*: central venous pressure, *IAP*: intra-abdominal pressure, *IVCCI*: inferior vena cava collapsibility index, *MV*: mechanical ventilation, *NIV*: non-invasive ventilation, *CPAP*: continuous positive airway pressure

During the study period, 419 patients were screened for eligibility, 239/419 (57.0%) of whom were primarily excluded. The reasons for exclusion were the lack of need for CVC insertion (*n* = 111/239, 46.4%), prolonged MV or NIV/CPAP support (*n* = 26/239, 10.9%), BMI ≥ 35 kg/m^2^ (*n* = 41/239, 17.2%), high IAP (*n* = 37/239, 15.5%), pregnancy (*n* = 3/239, 1.3%), or refusal to participate in the trial (*n* = 21/239, 8.8%). One hundred and eighty patients providing 540 pairs of CVP measures with their matched IVCCI values were included in the study. One hundred and twenty-one (67.2%) patients contributing 177 paired measures were randomly selected for the training cohort, and the other 59 (32.8%) for the validation cohort (Fig. 2).

### Characteristics of the study population

The mean ± SD age of the study cohort was 57.6 ± 13.0 years, with a female/male ratio of 77/103. Sixty-three (35.0%) patients were admitted to the ICU for medical reasons, 61 (33.9%) for postoperative care after cardiac surgery, and 35 (19.4%) for postoperative care after non-cardiac surgery. Sepsis or septic shock accounted for 21 (11.7%) of the admissions. The mean ± SD of the CVP and IVCCI was 7.5 ± 3.2 mmHg and 31.9 ± 14.3%, respectively. Of the 540 CVP measures, 118/540 (21.9%) were categorized as low (< 5 mmHg), 296/540 (54.8%) as normal (5–10 mmHg), and 126/540 (23.3%) as high (> 10 mmHg). The time lag between the acquisition of CVP and IVCCI measures was 3.7 ± 0.8 min. The characteristics of the whole study cohort as well as the training and validation subsets are detailed in Table 1.

**Table 1:** Characteristics of the study population

*Variable*	*Whole cohort (N* = *180)*	*Training cohort (n* = *121)*	*Validation cohort (n* = *59)*
Age (yr)	58 ± 13 (24–90)	57 ± 13 (24–89)	58 ± 14 (30–90)
Male sex	103/180 (57.2%)	74/121 (61.2%)	29/59 (49.2%)
Hypertension	87/180 (48.3%)	61/121 (50.4%)	26/59 (44.1%)
Diabetes mellitus	53/180 (29.4%)	39/121 (32.2%)	14/59 (23.7%)
Heart disease	81/180 (45.0%)	56/121 (46.3%)	25/59 (42.4%)
Chronic obstructive pulmonary disease	8/180 (4.4%)	4/121 (3.3%)	4/59 (6.8%)
Hypothyroidism	6/180 (3.3%)	2/121 (1.7%)	4/59 (6.8%)
Stroke	11/180 (6.1%)	9/121 (7.4%)	2/59 (3.4%)
Chronic liver disease	10/180 (5.6%)	6/121 (5.0%)	4/59 (6.8%)
Malignancy	17/180 (9.4%)	12/121 (9.9%)	5/59 (8.5%)
Dementia	6/180 (3.3%)	1/121 (0.8%)	5/59 (8.5%)
Chronic kidney disease	17/180 (9.4%)	11/121 (9.1%)	6/59 (10.2%)
Autoimmune disease	2/180 (1.1%)	2/121 (1.7%)	0/59 (0.0%)
Indication for ICU admission			
Medical indications	63/180 (35.0%)	40/121 (33.1%)	23/59 (39.0%)
Sepsis/Septic shock	21/180 (11.7%)	16/121 (13.2%)	5/59 (8.5%)
Postoperative care after cardiac surgery	61/180 (33.9%)	39/121 (32.2%)	22/59 (37.3%)
Postoperative care after non-cardiac surgery	35/180 (19.4%)	26/121 (21.5%)	9/59 (15.3%)
CVP_1_ (mmHg)	7.2 ± 3.6 (0.0–18.4)	6.8 ± 3.7 (0.0–18.4)	7.9 ± 3.1 (0.7–13.2)
CVP_2_ (mmHg)	7.6 ± 3.6 (0.0–19.9)	7.3 ± 3.8 (0.0–19.9)	8.4 ± 3.2 (2.9–15.4)
CVP_3_ (mmHg)	7.8 ± 3.4 (0.0–18.4)	7.7 ± 3.6 (0.0–17.6)	8.0 ± 3.1 (1.5–18.4)
Average of the three CVP measures	7.5 ± 3.2 (0.73–18.6)	7.3 ± 3.4 (0.7–18.6)	8.1 ± 2.8 (2.7–15.7)
Qualitative assessment of the CVP measures			
Low CVP (< 5 mmHg)	118/540 (21.9%)	93/363 (25.6%)	24/177 (13.6%)
Normal CVP (5–10 mmHg)	296/540 (54.8%)	188/363 (51.8%)	109/177 (61.6%)
High CVP (> 10 mmHg)	126/540 (23.3%)	82/363 (22.6%)	44/177 (24.9%)
IVCd_max1_ (cm)	1.9 ± 0.6 (0.6–3.1)	2.0 ± 0.6 (0.6–3.1)	1.9 ± 0.6 (0.7–3.1)
IVCd_max2_ (cm)	2.0 ± 0.5 (0.6–3.9)	2.1 ± 0.5 (0.7–3.9)	2.0 ± 0.5 (0.6–3.0)
IVCd_max3_ (cm)	2.0 ± 0.6 (0.8–3.6)	2.0 ± 0.6 (0.8–3.0)	2.0 ± 0.6 (0.9–3.6)
Average of the IVCd_max_ measures	2.00 ± 0.49 (0.87–3.28)	2.03 ± 0.50 (0.87–3.28)	1.95 ± 0.48 (0.97–2.97)
IVCd_min1_ (cm)	1.3 ± 0.6 (0.1–2.8)	1.3 ± 0.6 (0.1–2.8)	1.3 ± 0.6 (0.3–2.6)
IVCd_min2_ (cm)	1.4 ± 0.6 (0.2–3.4)	1.5 ± 0.6 (0.2–3.4)	1.4 ± 0.5 (0.3–2.7)
IVCd_min3_ (cm)	1.4 ± 0.6 (0.3–2.9)	1.4 ± 0.6 (0.3–2.9)	1.3 ± 0.5 (0.5–2.8)
Average of the IVCd_min_ measures	1.38 ± 0.49 (0.47–2.73)	1.39 ± 0.50 (0.47–2.73)	1.37 ± 0.46 (0.53–2.50)
IVCCI_1_ (%)	33.2 ± 18.2 (3.4–93.0)	34.3 ± 19.6 (3.4–93.0)	30.8 ± 14.8 (7.1–80.0)
IVCCI_2_ (%)	30.4 ± 15.9 (3.4–80.0)	31.0 ± 17.1 (3.4–80.0)	29.0 ± 13.0 (6.0–60.0)
IVCCI_3_ (%)	32.2 ± 14.9 (5.8–76.4)	32.0 ± 15.7 (6.5–76.4)	32.6 ± 13.3 (5.8–64.2)
Average of the IVCCI measures (%)	31.9 ± 14.3 (5.7–78.6)	32.46 ± 15.54 (5.70–78.63)	30.81 ± 11.31 (8.47–65.97)
Time lag between CVP and IVCCI acquisition (min)	3.7 ± 0.8 (2.3–5.0)	3.7 ± 0.8 (2.3–5.0)	3.6 ± 0.7 (2.3–5.0)

Data are presented as mean ± standard deviation (minimum–maximum) or proportion (percentage)

Subscripted numbers in relation to the CVP and IVC metrics denote the time point of the measure. *1*: time point 1 (07:00 h), *2*: time point 2 (15:00 h), *3*: time point 3 (23:00 h)

*CVP* central venous pressure, *IVCCI* inferior vena cava collapsibility index, *IVCd*_*max*_ inferior vena cava maximum diameter, *IVCd*_*min*_ inferior vena cava minimum diameter

### Test–retest and inter-observer reliability for the CVP and IVCCI measurements

The ICC for test–retest reliability regarding the CVP was.96 (95% CI:0.91–0.98) for Rater 1, and.97 (95% CI:0.93–0.99) for Rater 2. The ICC for inter-observer reliability was.91 (95% CI:0.77–0.96). As regards the IVCCI, the ICC for test–retest reliability was.99 (95% CI:0.97–0.99) for Rater 1, and.97 (95% CI:0.94–0.99) for Rater 2. The ICC for inter-observer reliability was.96 (95% CI:0.91–0.99).

### Correlation between the CVP and IVCCI and the discriminative value of the IVCCI

There was a moderate negative correlation between the CVP and IVCCI (Pearson’s *r* = –0.472, *P* < 0.001, *R*^*2*^ = 0.223) (Supplementary Fig. 1).

**Fig. 3:**
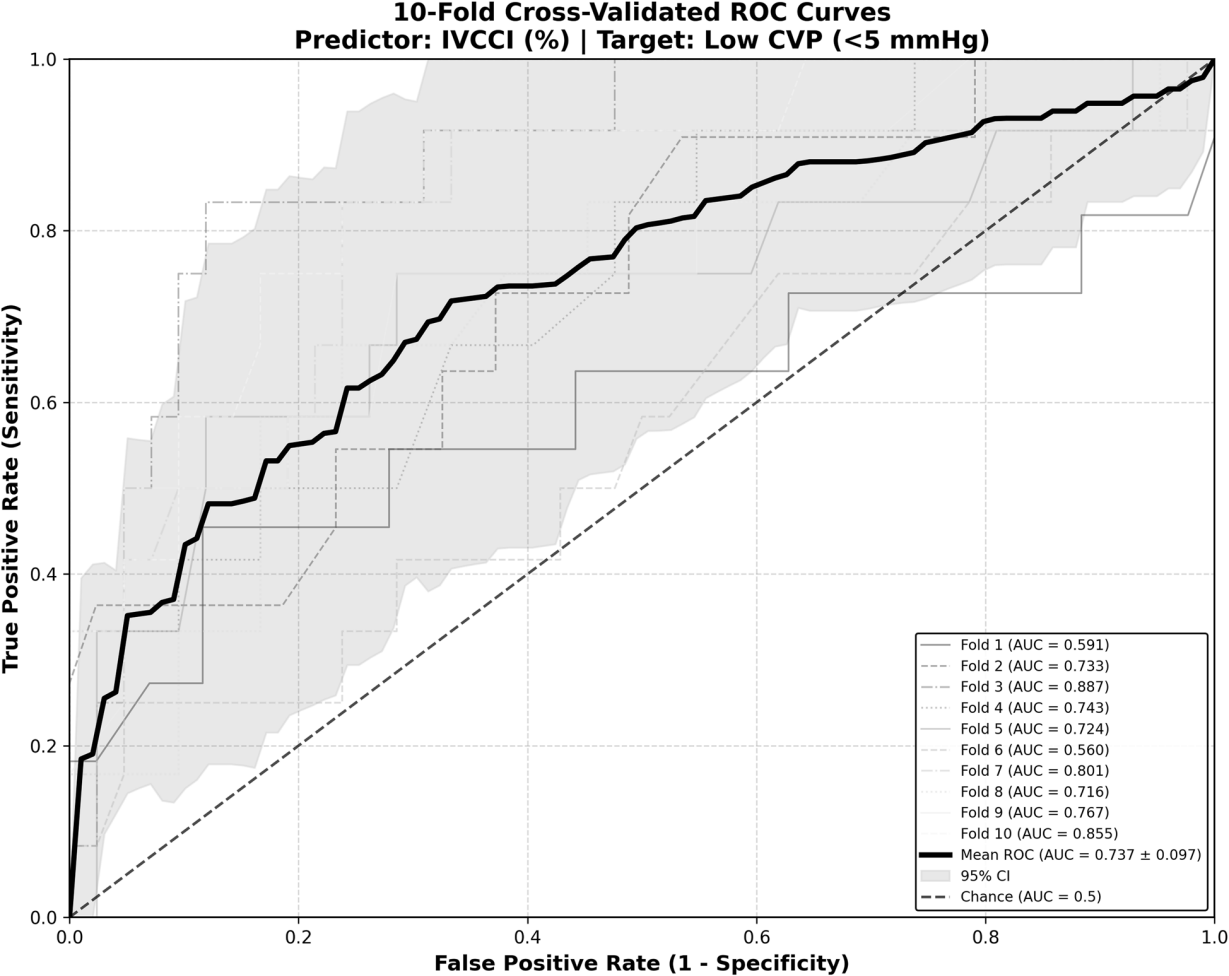
Cross-validated receiver-operating characteristic curves for prediction of low (< 5 cmH_2_O) central venous pressure using the inferior vena cava collapsibility index. Area under the curve =.737 (95% CI:.681–.790, *P* <.0001), overall accuracy = 71.5% (95% CI: 65.6%–78.9%), Cutoff ≥ 23% (95% CI: 19–28%). *CVP*: central venous pressure, *IVCCI*: inferior vena cava collapsibility index, *ROC*: receiver-operating characteristic

**Fig. 4:**
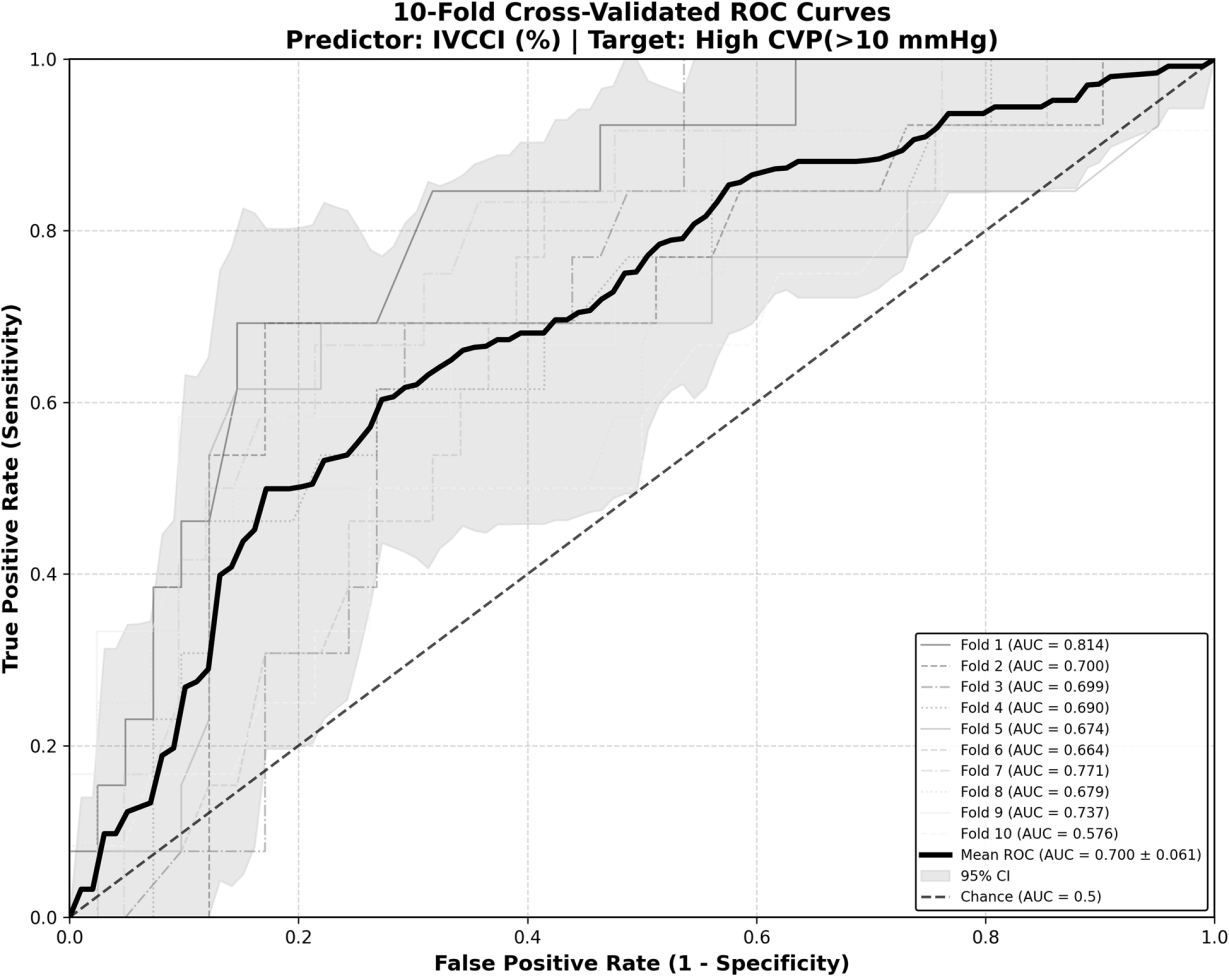
Cross-validated receiver-operating characteristic curves for prediction of high (> 10 cmH_2_O) central venous pressure using the inferior vena cava collapsibility index. Area under the curve =.700 (95% CI:.651–.757,* P* <.0001), overall accuracy = 75.2% (95% CI: 65.4–78.7%). Cutoff ≤ 30% (95% CI: 24–31%). *CVP*: central venous pressure, *IVCCI*: inferior vena cava collapsibility index, *ROC*: receiver-operating characteristic

The IVCCI demonstrated fair discriminative ability for identifying low CVP (< 5 mmHg), with an AUC of.737 (95% CI:0.681–0.790, *P* < 0.0001). An IVCCI ≥ 23% (95% CI: 19–28%) provided an overall accuracy of 71.5% (95% CI: 65.6–78.9%) (Fig. 3). Conversely, for identifying high CVP (> 10 mmHg), the IVCCI showed fair discriminative ability with an AUC of.700 (95% CI:0.651–0.757, *P* < 0.0001). An IVCCI ≤ 30% (95% CI: 24–31%) provided an overall accuracy of 75.2% (95% CI: 65.4–78.7%) (Fig. 4). These findings are consistent with the inverse relationship between IVCCI and CVP, where greater IVC collapsibility (higher IVCCI) reflects lower intravascular volume and lower CVP, whereas reduced collapsibility (lower IVCCI) reflects higher CVP. The details of the cross-validated ROC metrics are displayed in Supplementary Table 1.

### Fitting and selection of the training model

We fitted the training model using a Tweedie distribution with a log link function to resolve the issue of skewness and zero values of the CVP measures and opted for a parsimonious model including the intercept and IVCCI only, as this showed the best fit (QIC: 289.170, QICC: 284.763) (Supplementary Table 2). The model achieved convergence within four iterations with negligible change in coefficients after the second iteration (Supplementary Table 3). The parameter estimates of the training model are shown in Supplementary Table 4. The working correlation indicated a strong, positive correlation among repeated CVP measurements (*r* = 0.702), denoting consistent within-patient relationships over time and supporting the use of a GEE framework (Supplementary Table 5). The derived equation was as follows:

$$\mathrm{Log}(\mathrm{CVP}) \left(\mathrm{mmHg}\right) = 2.365 - 0.013 \times \mathrm{IVCCI} (\%)$$

The predicted CVP was calculated by exponentiating the equation as follows:

$$\text{Predicted CVP} \left(\mathrm{mmHg}\right) = \exp\left(2.365 - 0.013 \times \mathrm{IVCCI} (\%)\right)$$

This implies that for every 1 percentage point increase in the IVCCI, the predicted CVP decreases by approximately 1.3% (exp[− 0.013] = 0.987, 95% CI: 0.983–0.992).

### Testing the assumptions of the training model

There was no statistically significant correlation between the raw residuals and predicted CVP values (*r* = 0.036, *P* = 0.499), indicating that the residuals were independent of the fitted values (Supplementary Fig. 2). The frequency distribution of the raw residuals showed a mean value of 0.031 with moderate dispersion (SD = 3.34). The skewness coefficient of 0.391 indicated a mild right-tailed asymmetry but remained within the acceptable limits for normality (Supplementary Fig. 3 & Supplementary Table 6).

### Validation of the training model on the validation dataset

To assess the clinical performance of the training model, we validated the obtained rule on the hold-out (validation) dataset, in which the estimated marginal mean (EMM) CVP was 8.1 mmHg (95% CI: 7.4–8.8 mmHg) compared with 7.2 mmHg (95% CI: 6.7–7.9 mmHg) in the training sample (difference: 0.8 mmHg, 95% CI: –0.1–1.8 mmHg, *P* = 0.077). The EMM IVCCI was 30.8% (95% CI: 28.1–33.8%) versus 32.5% (95% CI: 29.8–35.3%) in either dataset, respectively (difference: –1.7%, 95% CI: –5.6–2.3%, *P* = 0.415).

The MAE was 2.13 mmHg (SD = 1.78 mmHg), with a range of 0.03 to 9.82 mmHg. This indicates that, on average, the model’s predictions were within approximately 2 mmHg of the measured CVP values. However, the distribution of absolute errors showed moderate positive skewness (skewness coefficient = 1.418), denoting that while most predictions were reasonably accurate, a small number of larger errors did occur (Supplementary Table 7).

The RMSE of 2.77 mmHg was slightly higher than the MAE (2.13 mmHg), reflecting the presence of occasional larger errors that were penalized more heavily by the squared error metric (Supplementary Table 8).

There was a moderate-to-strong positive correlation between the predicted and measured CVP values (*r* = 0.544, *P* < 0.001) (Supplementary Fig. 4), and the model predicted the CVP within ± 1 mmHg in 28.2%, within ± 2 mmHg in 58.2%, within ± 3 mmHg in 75.7%, and within ± 4 mmHg in 85.3% of measurements (Supplementary Table 9).

**Fig. 5:**
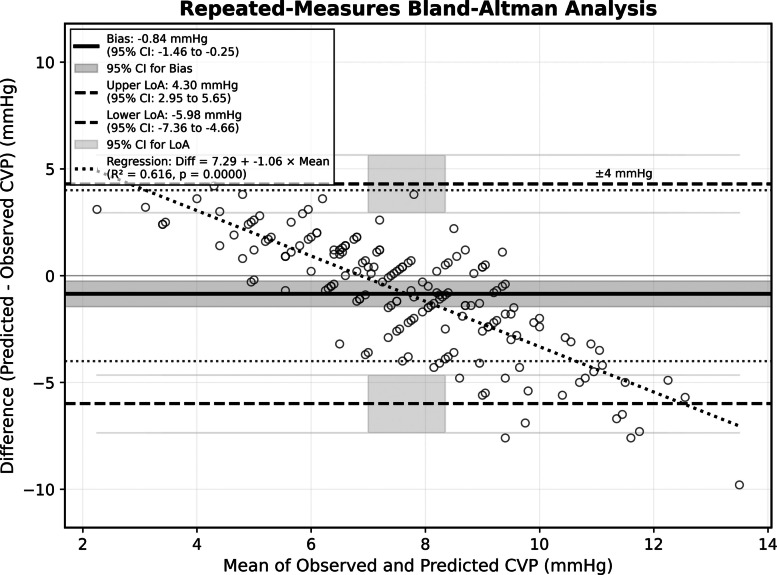
Repeated-measures Bland–Altman plot for agreement between predicted and observed CVP. The solid line represents the bias (mean: − 0.84 mmHg, 95% CI: − 1.46 to − 0.25 mmHg). The dashed lines represent the 95% limits of agreement (LoA: − 5.98 to 4.30 mmHg, 95% CI: − 7.36 to − 4.66 and 2.95 to 5.65 mmHg for the lower LoA and upper LoA, respectively). The dotted line represents the regression of differences on means (Difference = 7.29 − 1.062 × Mean, *R*^*2*^ =.616, *P* <.0001). The shaded areas represent the 95% confidence intervals for the limits of agreement. The thin dotted lines (gray) represent the prespecified maximum accepted difference of ± 4 mmHg (overall, 85.9% of predictions fell within the MAD of ± 4 mmHg). *95% CI*: 95% confidence interval, *CVP*: central venous pressure, *Diff*: difference, *LoA*: limits of agreement, *MAD*: maximum accepted difference, *R*^*2*^: coefficient of determination

The repeated-measures Bland–Altman analysis demonstrated a bias of –0.842 mmHg (95% CI: –1.458 to –0.255 mmHg), indicating a small but statistically significant overprediction of CVP by the model. The 95% LoA ranged from –5.981 mmHg (95% CI: –7.358 to –4.658 mmHg) to 4.298 mmHg (95% CI: 2.945 to 5.645 mmHg). Regression of the differences on the means revealed a significant proportional bias (slope = –1.062, 95% CI: –1.187 to –0.937, *P* < 0.001), with the model tending to over-predict at low CVP values and under-predict at high CVP values. The *R*^*2*^ was 0.616, indicating that the mean CVP explained 61.6% of the variance in the differences. Overall, 85.9% of predictions fell within the prespecified MAD of ± 4 mmHg (Fig. 5).

**Fig. 6:**
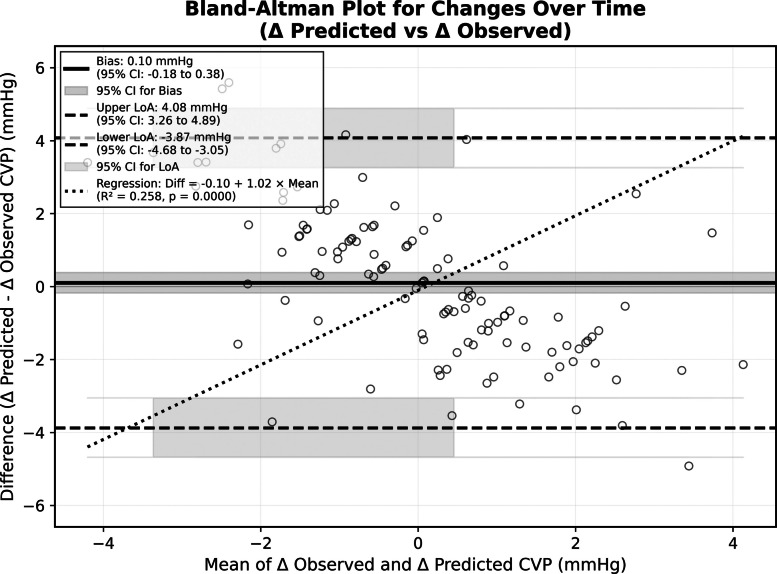
Repeated-measures Bland–Altman plot for agreement between the change in predicted CVP and change in observed CVP. The solid line represents the bias (mean: 0.10 mmHg, 95% CI: − 0.18 to 0.38 mmHg). The dashed lines represent the 95% limits of agreement (LoA: − 3.87 to 4.08 mmHg, 95% CI: − 4.68 to − 3.05 and 3.26 to 4.89 mmHg for the lower LoA and upper LoA, respectively). The dotted line represents the regression of differences on means (Difference = − 0.10 + 1.02 × Mean, R2 =.258, P .0001). 95% CI: 95% confi dence interval, CVP: central venous pressure, Diff : difference, Δ Observed: change in observed CVP, Δ Predicted: change in predicted CVP, LoA: limits of agreement, R2: coefficient of determination

To assess the model’s utility for trend monitoring, we evaluated the agreement between changes in predicted CVP and changes in measured CVP between the three consecutive time points. This analysis was conducted on 118 pairs of Δ Predicted-Δ Observed CVP measures. There was a moderate positive correlation between Δ Observed and Δ Predicted CVP (Pearson’s *r* = 0.508, *R*^*2*^ = 0.258, *P* < 0.001). Regression of the Δ Observed on Δ Predicted CVP yielded a slope of 1.023 (95% CI: 0.273–1.341, *P* < 0.001) and an intercept of –0.103 (95% CI: –0.479 to 0.704, *P* = 0.588), indicating that changes were reasonably well-calibrated with no significant systematic bias. The Bland–Altman analysis demonstrated a small bias of 0.101 mmHg (95% CI: –0.178 to 0.384), with 95% LoA ranging from –3.874 mmHg (95% CI: –4.680 to –3.055) to 4.076 mmHg (95% CI: 3.261 to 4.886). The model correctly predicted the direction of change in 65.3% of instances (Fig. 6).

### Calibration of the model on the validation sample

**Fig. 7:**
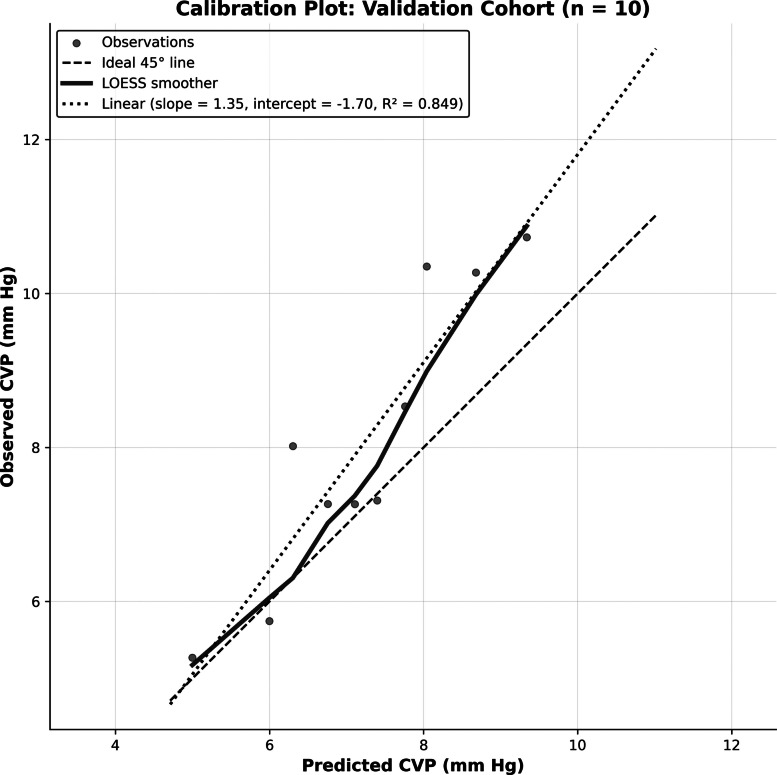
Calibration plot for the prediction rule. Predicted CVP values are grouped into ten deciles. The mean observed CVP (y-axis) is plotted against the mean predicted CVP (x-axis). The dashed line represents the line of identity (y = x), indicating perfect calibration (*i.e.*, ideal 45° line). The dotted line represents the linear regression line (*i.e.*, observed regressed on predicted CVP). The thick solid line represents the Local Regression Smoothing (LOESS) line. The model’s predictions are too conservative, underestimating high CVP values (calibration slope: 1.35, 95% CI: 1.70–1.81, *P* <.001), with no systematic bias (intercept: –1.70, 95% CI: –5.11–0.89, *P* =.283)

Linear regression of observed CVP on predicted CVP yielded a calibration slope of 1.35 (95% CI: 1.70–1.81, *P* < 0.001) and an intercept of –1.70 (95% CI: –5.11–0.89, *P* = 0.283). The calibration slope was significantly greater than 1.0, indicating that the model’s predictions are too conservative, underestimating high CVP values. The intercept was not significantly different from zero, suggesting no systematic bias across the entire observation range (Supplementary Table 10). These findings, combined with the non-linear pattern observed in the LOESS calibration plot (Fig. 7), indicated that the model may require non-linear recalibration to optimize its performance across the full CVP spectrum.

We attempted to apply a shrinkage factor of 0.731 (reciprocal of calibration slope) to the prediction rule to improve its performance [39]. Linear regression of observed CVP on predicted CVP yielded a calibration slope of 1.65 (95% CI: –0.52 to 2.23, *P* < 0.001) and an intercept of –5.15 (95% CI: –9.79 to 1.08, *P* = 0.034), denoting worsening of the predictions with a statistically significant systematic overprediction bias. The confidence interval for the slope also widened substantially from a range of 0.110 to 2.742, indicating greater uncertainty. These findings implied that the applied shrinkage factor was unhelpful, in effect potentially harmful, and that the model could benefit from non-linear recalibration rather than simple linear shrinkage.

## Discussion

### Principal findings and their clinical implications

In the present study, we developed and validated a prediction rule to estimate the CVP non-invasively from the IVCCI. The proposed model showed promising accuracy with an MAE of approximately 2 mmHg and > 75% of predictions falling within ± 3 mmHg of the true CVP. In effect, the moderate-to-strong correlation between predicted and observed CVP implied that the model captured an important proportion of the variance in the true CVP values of the order of 30%. However, a scrupulous assessment of the model’s performance revealed that it may have some weaknesses. Firstly, the moderately positive skewness coefficient of 1.418 for the absolute errors and the RMSE of about 2.8 mmHg indicated the presence of occasional larger errors in some subgroups. In fact, the agreement analysis provided noteworthy insights into this behavior, where 95% of the predictions agreed with the true measures within rather lenient limits that spanned the range from about –6 to + 4 mmHg, with the additional finding that the model tended to systematically over-predict at low CVP values and vice versa, a pattern that was also attested to by the calibration plot that indicated while the model was well-calibrated for the average range, it failed to adequately capture extremely low or high values, which are often the most relevant targets for a clinician, warranting special caution with the interpretation of the model’s estimations.

Despite these shortcomings, the model may still be regarded as a clinically helpful tool, where the clinical utility of the model is best weighed from a pragmatic perspective. With a substantial majority of instances exceeding 85% in which the model’s predictions were within a prespecified clinically acceptable range of ± 4 mmHg, the present rule can be suggested for the routine monitoring and initial triage in a broad patient population; the model performs adequately. However, with an anticipated proportional bias and nearly 15% of predictions falling outside the clinically acceptable range, the model, in its current linear form, should not be recommended as a standalone, definitive diagnostic tool, particularly in situations where extreme CVP values are expected, such as in critically ill patients with severe hypovolemia or overt fluid overload. Its value may lie more in serial trend monitoring. Of note in this context, the model exhibited good agreement for trend monitoring over time, where it correctly predicted the direction of change in about 65% of instances, which, while not perfect, could serve as a useful adjunctive signal for clinicians. This is a significant finding, as tracking the directional change and magnitude of CVP in response to therapy (e.g., fluid boluses or diuretics) is often more clinically actionable than a single isolated measurement.

To frame all this within a realistic context, it should not be overstressed that the apparent proportional bias and the model’s compression of predictions at clinical extremes warrant caution. Therefore, the model is not to replace standard invasive CVP measurement in high-stakes scenarios but could be a valuable screening or trend-monitoring tool in less critical settings or as an additional parameter in a multivariable clinical assessment. In fact, the failure of our attempt at linear recalibration to bring this shortcoming into reconciliation highlights the need for non-linear recalibration to correct the model’s inherent deficiencies and improve its accuracy across the entire spectrum of CVP values. As such, the present study provides a robust foundation and a clear roadmap for these necessary refinements, moving the model closer to a clinically reliable, non-invasive CVP prediction tool.

### Literature gap addressed by the present study

The present study fills an important literature gap. While several trials investigated the relationship between the CVP and IVCCI [7–17] or managed to determine IVCCI cut-offs to identify patients with abnormally high or low CVP values [8, 10, 13–17], not many trials offered a prediction rule for estimating the CVP from ultrasound-measured IVC dynamics. For instance, one such study fitted an equation to calculate the CVP from the IVC dynamics in mechanically ventilated patients but was not formally validated [11]. Another equation estimated the RAP from the IVCCI in adults with CHD who underwent cardiac catheterization [12]. Though that study was validated on an independent hold-out sample, it had major methodological limitations. As perceived from the reported methods, a time gap of up to 7 days was allowed between measuring RAP during cardiac catheterization and acquiring the IVCCI by transthoracic echocardiography, which invalidates the real-time relation between both parameters. This major shortcoming, combined with the retrospective nature of the trial and selective eligibility criteria for the study cohort, limits the validity and generalizability of that equation as a clinically useful tool for real-time estimation of the CVP.

### Strengths of the study

The present study has several strengths. *First*, the development of a simple, validated tool for the non-invasive estimation of CVP addresses a relevant need of clinicians caring for critically ill patients, who by far represent the largest patient population requiring central venous cannulation for optimization of their fluid and hemodynamic management. *Second*, the study employed robust statistical methods, including a repeated-measures design with multiple measurements per patient, substantially increasing the effective sample size and statistical power, while enabling the assessment of temporal hemodynamic variability. Of note, the use of a GEE model [29] with a Tweedie distribution [30] and log link [31] appropriately accounted for the zero-inflated, skewed distribution of data and within-subject correlation inherent in repeated measures of CVP in the real world. *Third*, we employed a rigorous methodology for validation of the suggested rule, utilizing a patient-level approach to splitting the sample to prevent data leakage and ensure that all observations from the same patient were kept together in a single subset. *Fourth*, the comprehensive performance assessment encompassed both cross-sectional agreement and the ability to track changes over time, including the use of a clustered bootstrap for repeated-measures Bland–Altman analysis to avoid artificially narrow LoA [35, 36]. *Fifth*, a relevant aspect in the present study is that it included a diverse cohort of medical, surgical, as well as critically ill patients, an attribute that should be viewed as a merit rather than a shortcoming. Critically, this could be claimed to foster the model’s generalizability as a versatile, all-purpose tool. Of particular relevance in this regard is that we did explore whether inclusion of more candidate predictors such as the indication for ICU admission, otherwise denoting the patient’s category, or time of measurement as fixed factors in the predictive model would significantly influence the equation’s parameters and demonstrated that these factors did not improve the model’s goodness of fit, supporting the selection of a simple model comprising the IVCCI and intercept only.

### Study limitations

On the other hand, several limitations should be acknowledged. *First*, the present study comprised spontaneously breathing adult patients from a single center, which may limit its applicability to other healthcare systems or patient populations, such as children or mechanically ventilated patients. Of note are patients on MV, in whom IVC dynamics differ significantly due to the positive intrathoracic pressure [6]. Thus, although we performed robust internal validation using a split-sample approach, external validation in independent, multicenter cohorts is indispensable before the model can be adopted for general-purpose clinical applications [40]. *Second*, the IVCCI measurement is both operator- and mode-dependent [41, 42] and may be influenced by image quality or patient body habitus. Additionally, owing to the intolerance to assuming the supine position by some patients, we had to obtain the CVP and IVC measurements in a semi-sitting position. However, it is a usual encounter in the real world that invasive measures have to be taken in non-standard positions and there is compelling evidence that the IVC metrics are minimally altered when changing from the supine to the upright position [23], as are the CVP measures if the phlebostatic axis is set where the midaxillary line intersects the 4th intercostal space, regardless of the position assumed by the patient [22]. *Third*, the IVCCI is a dynamic variable that is susceptible to rapid changes in response to respiratory effort and clinical interventions [5]. Therefore, while we standardized the acquisition of CVP and IVCCI measures within a narrow time window of ≤ 5 min, we cannot discount the possibility that some of the observed variability did reflect true physiological changes rather than random measurement error.

### Future directions

As potential directives for future research, external validation of the proposed rule in independent, multicenter cohorts across diverse clinical settings and patient populations is of paramount priority to confirm its generalizability. Another likely area is recalibrating the model on an external cohort to enhance its accuracy. For this purpose, more elaborate methods, *e.g*., non-linear or multivariable modeling incorporating other relevant clinical predictors, may be of value.

## Conclusions

This study developed and validated a prediction model for estimating the CVP non-invasively from IVCCI in spontaneously breathing critically ill adults. The model demonstrated a promising performance, with a mean absolute error of approximately 2 mmHg, over three-quarters of predictions falling within ± 3 mmHg of true CVP values, and about two-thirds of directional changes correctly predicted. However, the rather wide LoA and the model’s tendency to over- or under-predict at low or high CVP values, respectively, indicate that the model is not precise enough to substitute invasive CVP measurement for high-stakes clinical decisions. Nonetheless, the proposed rule may be useful for screening and triage purposes to identify patients with clearly low or high CVP who may benefit from further invasive monitoring. Intuitively, external validation in independent, multicenter cohorts is fundamental before the model can be recommended for regular clinical use. Recalibration of the model on larger external cohorts or attempting to enhance its performance by applying more complex modeling methods may also be proposed as possible directives for future research.

## Supplementary Information

Additional file 1: Supplementary Tables and Figures.

## Data Availability

The datasets collected and/or analyzed during the current study are not publicly available due to patient confidentiality and ethical restrictions but are available from the corresponding author on reasonable request.

## Abbreviations

AUC: Area under the curve
BMI: Body mass index
CHD: Congenital heart disease
CI: Confidence intervals
CITL: Calibration-in-the-large
CPAP: Continuous positive airway pressure
CVC: Central venous catheter
CVP: Central venous pressure
GEE: Generalized estimating equations
IAP: Intra-abdominal pressure
ICC: Intraclass correlation coefficient
ICU: Intensive care unit
IJV: Internal jugular vein
IVC: Inferior vena cava
IVCCI: Inferior vena cava collapsibility index
LoA: Limits of agreement
LOESS: Local regression smoothing
MAD: Maximum accepted difference
MAE: Mean absolute error
MV: Mechanical ventilation
NIV: Non-invasive ventilation
QIC: Quasi-likelihood under independence model criterion
QICC: Corrected quasi-likelihood under independence model criterion
RAP: Right atrial pressure
RMSE: Root mean square error
ROC: Receiver-operating characteristic
SD: Standard deviation
TRIPOD: Transparent Reporting of a multivariable prediction model for Individual Prognosis Or Diagnosis

## References

[1] Persichini R, Lai C, Teboul JL, Adda I, Guérin L, Monnet X (2022) Venous return and mean systemic filling pressure: physiology and clinical applications. Crit Care 26:150 35610620 10.1186/s13054-022-04024-x PMC9128096

[2] Pinelli F, Pittiruti M, Annetta MG, Barbani F, Bertoglio S, Biasucci DG, Bolis D, Brescia F, Capozzoli G, D’Arrigo S, Deganello E, Elli S, Fabiani A, Fabiani F, Gidaro A, Giustivi D, Iacobone E, La Greca A, Longo F, Lucchini A, Marche B, Romagnoli S, Scoppettuolo G, Selmi V, Vailati D, Villa G, Pepe G (2025) A GAVeCeLT consensus on the indication, insertion, and management of central venous access devices in the critically ill. J Vasc Access 26:1096–1114 39097780 10.1177/11297298241262932

[3] Babaie S, Behzad A, Mohammadpour M, Reisi M (2018) A comparison between the bedside sonographic measurements of the inferior vena cava indices and the central venous pressure while assessing the decreased intravascular volume in children. Adv Biomed Res 7:97 30050885 10.4103/abr.abr_213_17 PMC6036785

[4] Ter Schiphorst B, Bourel C, Durand A, Ternynck C, Onimus T, Howsam M, Pierre A, De Jonckheere J, Favory R, Preau S (2026) Validation of the inferior vena cava collapsibility as a predictive marker of fluid responsiveness in spontaneously breathing patients. Sci Rep 16:15678 41927624 10.1038/s41598-026-41826-3 PMC13187124

[5] Kircher BJ, Himelman RB, Schiller NB (1990) Noninvasive estimation of right atrial pressure from the inspiratory collapse of the inferior vena cava. Am J Cardiol 66:493–496 2386120 10.1016/0002-9149(90)90711-9

[6] Jue J, Chung W, Schiller NB (1992) Does inferior vena cava size predict right atrial pressures in patients receiving mechanical ventilation? J Am Soc Echocardiogr 5:613–619 1466886 10.1016/s0894-7317(14)80327-1

[7] Kumar A, Bharti AK, Hussain M, Kumar S, Kumar A (2024) Correlation of internal jugular vein and inferior vena cava collapsibility index with direct central venous pressure measurement in critically-ill patients: an observational study. Indian J Crit Care Med 28:595–600 39130396 10.5005/jp-journals-10071-24741 PMC11310668

[8] Garcia RU, Meert KL, Safa R, Aggarwal S (2021) Inferior vena cava collapsibility index to assess central venous pressure in perioperative period following cardiac surgery in children. Pediatr Cardiol 42:560–568 33481045 10.1007/s00246-020-02514-9

[9] Ilyas A, Ishtiaq W, Assad S, Ghazanfar H, Mansoor S, Haris M, Qadeer A, Akhtar A (2017) Correlation of IVC diameter and collapsibility index with central venous pressure in the assessment of intravascular volume in critically ill patients. Cureus 9:e1025 28348943 10.7759/cureus.1025 PMC5346017

[10] Jassim HM, Naushad VA, Khatib MY, Chandra P, Abuhmaira MM, Koya SH, Ellitthy MSA (2019) IJV collapsibility index vs IVC collapsibility index by point of care ultrasound for estimation of CVP: a comparative study with direct estimation of CVP. Open Access Emerg Med 11:65–75 31040727 10.2147/OAEM.S176175 PMC6452797

[11] Karacabey S, Sanri E, Guneysel O (2016) A non-invasive method for assessment of intravascular fluid status: inferior vena cava diameters and collapsibility index. Pak J Med Sci 32:836–840 27648024 10.12669/pjms.324.10290 PMC5017087

[12] Egbe AC, Connolly HM, Pellikka PA, Anderson JH, Miranda WR (2022) Role of inferior vena cava dynamics for estimating right atrial pressure in congenital heart disease. Circ Cardiovasc Imaging 15:e014308 36126125 10.1161/CIRCIMAGING.122.014308 PMC9504388

[13] Mulder TA, Becude L, Lopez Matta JE, van den Hout WB, van Westerloo DJ, Bauer MP (2024) The SONIC CENTRAL study: central venous pressure in hospitalized patients – accuracy of physical examination and point-of-care ultrasonography of the jugular vein and inferior vena cava. CHEST Crit Care 2:100091

[14] Nagdev AD, Merchant RC, Tirado-Gonzalez A, Sisson CA, Murphy MC (2010) Emergency department bedside ultrasonographic measurement of the caval index for noninvasive determination of low central venous pressure. Ann Emerg Med 55:290–295 19556029 10.1016/j.annemergmed.2009.04.021

[15] Premkumar M, Rangegowda D, Kajal K, Khumuckham JS (2019) Noninvasive estimation of intravascular volume status in cirrhosis by dynamic size and collapsibility indices of the inferior vena cava using bedside echocardiography. JGH Open 12(3):322–328 10.1002/jgh3.12166 PMC6684769 31406926

[16] Worapratya P, Anupat S, Suwannanon R, Wuthisuthimethawee P (2014) Correlation of caval index, inferior vena cava diameter, and central venous pressure in shock patients in the emergency room. Open Access Emerg Med 6:57–62 27147880 10.2147/OAEM.S60307 PMC4753986

[17] Prekker ME, Scott NL, Hart D, Sprenkle MD, Leatherman JW (2013) Point-of-care ultrasound to estimate central venous pressure: a comparison of three techniques. Crit Care Med 41(3):833–841 23318493 10.1097/CCM.0b013e31827466b7

[18] Chen CY, Zhou Y, Wang P, Qi EY, Gu WJ (2020) Elevated central venous pressure is associated with increased mortality and acute kidney injury in critically ill patients: a meta-analysis. Crit Care 24:80 32138764 10.1186/s13054-020-2770-5 PMC7059303

[19] Collins GS, Reitsma JB, Altman DG, Moons KG (2015) Transparent reporting of a multivariable prediction model for individual prognosis or diagnosis (TRIPOD): the TRIPOD statement. Ann Intern Med 162:55–63 25560714 10.7326/M14-0697

[20] See KC, Tayebi S, Sum CL, Phua J, Stiens J, Wise R, Mukhopadhyay A, Malbrain MLNG (2023) Feasibility analysis of a novel non-invasive ultrasonographic method for the measurement of intra-abdominal pressure in the intensive care unit. J Clin Monit Comput 37:135–139 10.1007/s10877-023-01024-2 37133628

[21] Sjödin C, Sondergaard S, Johansson L (2019) Variability in alignment of central venous pressure transducer to physiologic reference point in the intensive care unit-A descriptive and correlational study. Aust Crit Care 32:213–217 29866610 10.1016/j.aucc.2018.05.001

[22] Avellan S, Uhr I, McKelvey D, Sondergaard S (2017) Identifying the position of the right atrium to align pressure transducer for CVP: spirit level or 3D electromagnetic positioning? J Clin Monit Comput 31:943–949 27510178 10.1007/s10877-016-9918-5

[23] Panebianco NL, Shofer F, Cheng A, Fischer J, Cody K, Dean AJ (2014) The effect of supine versus upright patient positioning on inferior vena cava metrics. Am J Emerg Med 32:1326–1329 25256269 10.1016/j.ajem.2014.07.036

[24] Li Z, McKeague IW (2013) Power and sample size calculations for generalized estimating equations via local asymptotics. Stat Sin 23:231–250 24478568 10.5705/ss.2011.081 PMC3903421

[25] Liu J, Colditz GA (2017) Optimal design of longitudinal data analysis using generalized estimating equation models. Biom J 59:315–330 27878852 10.1002/bimj.201600107 PMC5575779

[26] Schick A (2001) Sample splitting with Markov chains. Bernoulli 7:33–61

[27] Singh V, Pencina M, Einstein AJ, Liang JX, Berman DS, Slomka P (2021) Impact of train/test sample regimen on performance estimate stability of machine learning in cardiovascular imaging. Sci Rep 11:14490 34262098 10.1038/s41598-021-93651-5 PMC8280147

[28] Steyerberg EW, Harrell FE (2016) Prediction models need appropriate internal, internal-external, and external validation. J Clin Epidemiol 69:245–247 25981519 10.1016/j.jclinepi.2015.04.005 PMC5578404

[29] Liang KY, Zeger SL (1986) Longitudinal data analysis using generalized linear models. Biometrika 73:13–22

[30] Kurz CF (2017) Tweedie distributions for fitting semicontinuous health care utilization cost data. BMC Med Res Methodol 17:171 29258428 10.1186/s12874-017-0445-y PMC5735804

[31] Box GEP, Cox DR (1964) An analysis of transformations. J R Stat Soc Ser B Stat Methodol 26:211–252

[32] White H (1980) A heteroskedasticity-consistent covariance matrix estimator and a direct test for heteroskedasticity. Econometrica 48:817–838

[33] Nelder JA, Wedderburn RWM (1972) Generalized linear models. J R Stat Soc Ser A 135:370–384

[34] Pan W (2001) Akaike’s information criterion in generalized estimating equations. Biometrics 57:120–125 11252586 10.1111/j.0006-341x.2001.00120.x

[35] Bland JM, Altman DG (2007) Agreement between methods of measurement with multiple observations per individual. J Biopharm Stat 17:571–582 17613642 10.1080/10543400701329422

[36] Parker RA, Weir CJ, Rubio N, Rabinovich R, Pinnock H, Hanley J, McCloughan L, Drost EM, Mantoani LC, MacNee W, McKinstry B (2016) Application of mixed effects limits of agreement in the presence of multiple sources of variability: exemplar from the comparison of several devices to measure respiratory rate in COPD patients. PLoS One 11:e0168321 27973556 10.1371/journal.pone.0168321 PMC5156413

[37] Zou GY (2013) Confidence interval estimation for the Bland-Altman limits of agreement with multiple observations per individual. Stat Methods Med Res 22:630–642 21705434 10.1177/0962280211402548

[38] Ludbrook J (2010) Confidence in Altman-Bland plots: a critical review of the method of differences. Clin Exp Pharmacol Physiol 37:143–149 19719745 10.1111/j.1440-1681.2009.05288.x

[39] Van Calster B, McLernon DJ, van Smeden M, Wynants L, Steyerberg EW, Topic Group ‘Evaluating diagnostic tests and prediction models’ of the STRATOS initiative (2019) Calibration: the Achilles heel of predictive analytics. BMC Med 17:230 31842878 10.1186/s12916-019-1466-7 PMC6912996

[40] Bleeker SE, Moll HA, Steyerberg EW, Donders AR, Derksen-Lubsen G, Grobbee DE, Moons KG (2003) External validation is necessary in prediction research: a clinical example. J Clin Epidemiol 56:826–832 14505766 10.1016/s0895-4356(03)00207-5

[41] Fields JM, Lee PA, Jenq KY, Mark DG, Panebianco NL, Dean AJ (2011) The interrater reliability of inferior vena cava ultrasound by bedside clinician sonographers in emergency department patients. Acad Emerg Med 18:98–101 21414063 10.1111/j.1553-2712.2010.00952.x

[42] Finnerty NM, Panchal AR, Boulger C, Vira A, Bischof JJ, Amick C, Way DP, Bahner DP (2017) Inferior vena cava measurement with ultrasound: what is the best view and best mode? West J Emerg Med 18:496–501 28435502 10.5811/westjem.2016.12.32489 PMC5391901

