# Supplementary material for: Development and validation of a proposed rule for estimating central venous pressure from inferior vena cava dynamics: a clinical prediction model study
Source: J Anesth Analg Crit Care. 2026 Jul 29;6:104. doi: 10.1186/s44158-026-00438-z (PMC13419015; doi:10.1186/s44158-026-00438-z)
Supplement: Supplementary file 1 — Additional file 1: Supplementary Tables and Figures. [file 44158_2026_438_MOESM1_ESM.docx]

Journal of Anesthesia, Analgesia and Critical Care

Supplementary Materials

Development and Validation of a Proposed Rule for Estimating Central Venous Pressure from Inferior Vena Cava Dynamics: A Clinical Prediction Model Study

Sameh M. HAKIM, <https://orcid.org/0000-0003-1900-0353>; ^1^ * Nermeen S. AHMED, <https://orcid.org/0009-0001-5253-6021>; ^2^ Sahar M. TALAAT, https:/0000-0001-6624-3345; ^1^ Sana F WASFY, https://: 0000-0002-9437-6644; ^3^ Adham M. HAGGAG, https:/0000-0002-7069-6707 ^3^

^1^ Professor, ^2^ Assistant Lecturer, ^3^ Assistant Professor, Department of Anesthesiology, Intensive Care, and Pain Management, Ain Shams University Faculty of Medicine, Cairo, Egypt

* Corresponding Author: Sameh M. Hakim: Professor, 15 Gamal Noah Street, Heliopolis, Almaza, Postal Code 11341, Cairo, Egypt; Email: [hakimsm@med.asu.edu.eg](mailto:hakimsm@med.asu.edu.eg), Telephone: +20226900073, Fax: +20226900073





Supplementary Figure 1. Scatter plot illustrating the correlation between the central venous pressure and inferior vena cava collapsibility index. *CVP*: central venous pressure, *IVCCI*: inferior vena cava collapsibility index, *R^2^*: coefficient of determination.

Supplementary Table 1. K-Fold cross-validated receiver-operating characteristic curve analysis for the discriminative value of the inferior vena cava collapsibility.

|  | Predicted outcome | |
| --- | --- | --- |
|  | Low CVP | High CVP |
| ROC Metric | Value (95% CI) | Value (95% CI) |
| AUC | .737 (.681–.790) | .700 (.651–.757) |
| IVCCI Cutoff | 23% (19–28%) | 30% (24–31%) |
| Sensitivity | 66.9% (52.6–79.7%) | 56.3% (49.7–75.0%) |
| Specificity | 72.7% (62.9–84.2%) | 80.9% (63.9–84.8%) |
| Youden Index | .397 (.317–.493) | .373 (.295–.476) |
| PPV | 40.7% (335–529%) | 47.3% (35.1–55.1%) |
| NPV | 88.7% (852–92.7%) | 85.9% (82.9–90.5%) |
| Accuracy | 71.5% (65.6–78.9%) | 75.2% (65.4–78.7%) |

*95% CI*: 95% confidence interval, *AUC*: area under the curve, *CVP*: central venous pressure, *IVCCI*: inferior vena cava collapsibility index, *NPV*: negative predictive value, *PPV*: positive predictive value, *ROC*: receiver-operating characteristic.

Supplementary Table 2. Goodness-of-fit statistics for five candidate predictive models

| Model | Predictors | QIC | QICC |
| --- | --- | --- | --- |
| Model 1 | (Intercept), IVCCI (%) | 289.170 | 284.763 |
| Model 3 | (Intercept), IVCCI (%), Time | 288.776 | 287.367 |
| Model 2 | (Intercept), IVCCI (%), Indication for ICU admission | 298.530 | 287.521 |
| Model 4 | (Intercept), IVCCI (%), Time, Indication for ICU admission | 298.018 | 290.067 |
| Model 5 | (Intercept), IVCCI (%), Time, Indication for ICU admission, Indication for ICU admission * IVCCI (%) | 313.419 | 294.554 |

Dependent Variable: CVP (mmHg).

*CVP*: central venous pressure, *ICU*: intensive care unit, *IVCCI*: inferior vena cava collapsibility index, *QIC*: quasi-likelihood under the independence criterion, *QICC*: corrected quasi-likelihood under the independence criterion.

Supplementary Table 3. Iteration history of the training model

| Iteration | Update Type | Parameter | | |
| --- | --- | --- | --- | --- |
|  |  | (Intercept) | IVCCI (%) | (Scale) |
| 0 | Initial | 2.428626 | -.014753 | .737937 |
| 1 | Scoring | 2.365535 | -.012723 | .737937 |
| 2 | Scoring | 2.365076 | -.012701 | .737937 |
| 3 | Scoring | 2.365069 | -.012701 | .737937 |
| 4 | Scoring^a^ | 2.365069 | -.012701 | .737937 |

Dependent Variable: CVP (mmHg).

Model: (Intercept), IVCCI (%).

^a^. All convergence criteria are satisfied.

*CVP*: central venous pressure, *IVCCI*: inferior vena cava collapsibility index.

Supplementary Table 4. Parameter estimates of the training model

| Parameter | B | SE | 95% CI | | Hypothesis Test | | | Exp(B) | 95% CI for Exp(B) | |
| --- | --- | --- | --- | --- | --- | --- | --- | --- | --- | --- |
|  |  |  | Lower | Upper | Wald *χ^2^* | df | *P* value |  | Lower | Upper |
| (Intercept) | 2.365 | 0.078 | 2.212 | 2.518 | 918.453 | 1 | <.001 | 10.645 | 9.135 | 12.404 |
| IVCCI (%) | -0.013 | 0.003 | -0.018 | -0.008 | 25.668 | 1 | <.001 | 0.987 | 0.983 | 0.992 |
| (Scale) | 0.738 |  |  |  |  |  |  |  |  |  |

Dependent Variable: CVP (mmHg).

Model: (Intercept), IVCCI (%).

*95% CI*: 95% confidence interval, *B*: regression coefficient, *CVP*: central venous pressure, *df*: degrees of freedom, *Exp(B)*: exponentiated coefficient, *IVCCI*: inferior vena cava collapsibility index, *SE*: standard error, *χ^2^*: chi-squared statistic.

Supplementary Table 5. Working correlation matrix of the training model

| Measurement | Measurement | | |
| --- | --- | --- | --- |
|  | CVP_1_ | CVP_2_ | CVP_3_ |
| CVP_1_ | 1 | .702 | .702 |
| CVP_2_ | .702 | 1 | .702 |
| CVP_3_ | .702 | .702 | 1 |

Dependent Variable: CVP (mmHg).

Model: (Intercept), IVCCI (%).

*CVP*: central venous pressure, *IVCCI*: inferior vena cava collapsibility index.

Subscripted numbers in relation to the CVP and IVC metrics denote the time point of the measure. *1*: time point 1 (07:00 h), *2*: time point 2 (15:00 h), *3*: time point 3 (23:00 h).





Supplementary Figure 2. Scatter plot illustrating the correlation between the raw residuals and fitted central venous pressure values for the training model.





Supplementary Figure 3. Histogram illustrating the frequency distribution of the raw residuals of the training model.

Supplementary Table 6. Descriptive statistics for the raw residuals of the training model

|  | N | Minimum | Maximum | Mean | SD | Skewness coefficient | SE of skewness coefficient |
| --- | --- | --- | --- | --- | --- | --- | --- |
| Raw Residual (mmHg) | 363 | -8.880 | 13.075 | 0.031 | 3.338 | 0.391 | 0.128 |
| Valid N (listwise) | 363 |  |  |  |  |  |  |

*N*: number, *SD*: standard deviation, *SE*: standard error, *Var.*: variance.

Supplementary Table 7. Clinical performance of the training model as tested on the validation sample: Descriptive statistics of the absolute error and calculation of the mean absolute error (MAE)

|  | N | Minimum | Maximum | Mean (MAE) | SD | Skewness coefficient | SE of skewness coefficient |
| --- | --- | --- | --- | --- | --- | --- | --- |
| Absolute error (mmHg) | 177 | 0.025 | 9.822 | 2.134 | 1.779 | 1.418 | 0.183 |
| Valid N (listwise) | 177 |  |  |  |  |  |  |

*MAE*: mean absolute error, *N*: number, *SD*: standard deviation, *SE*: standard error.

Supplementary Table 8. Clinical performance of the training model as tested on the validation sample: Descriptive statistics of the squared error and calculation of the root mean square error (RMSE)

|  | N | Minimum | Maximum | Mean (MSE) | SD | Skewness coefficient | SE of skewness coefficient | RMSE |
| --- | --- | --- | --- | --- | --- | --- | --- | --- |
| Squared error (mmHg^2^) | 177 | 0.0006 | 96.472 | 7.699 | 12.893 | 3.408 | 0.183 | 2.7748 |
| Valid N (listwise) | 177 |  |  |  |  |  |  |  |

*MSE:* mean squared error*, N*: number, *RMSE*: root mean square error, *SD*: standard deviation, *SE*: standard error.





Supplementary Figure 4. Clinical performance of the training model as tested on the validation sample: Scatter plot illustrating the correlation between the predicted and observed central venous pressure values. *CVP*: central venous pressure, *R^2^*: coefficient of determination.

Supplementary Table 9. Clinical performance of the training model as tested on the validation sample: Percentage of measures predicted within clinically acceptable ranges

| Accuracy Level | Error Range | Frequency (n) | Percent (%) | Valid Percent (%) | Cumulative Percent (%) |
| --- | --- | --- | --- | --- | --- |
| ±1 mmHg | > ±1 mmHg | 127 | 71.8 | 71.8 | 71.8 |
|  | ≤ ±1 mmHg | 50 | 28.2 | 28.2 | 100.0 |
| ±2 mmHg | > ±2 mmHg | 74 | 41.8 | 41.8 | 41.8 |
|  | ≤ ±2 mmHg | 103 | 58.2 | 58.2 | 100.0 |
| ±3 mmHg | > ±3 mmHg | 43 | 24.3 | 24.3 | 24.3 |
|  | ≤ ±3 mmHg | 134 | 75.7 | 75.7 | 100.0 |
| ±4 mmHg | > ±4 mmHg | 26 | 14.7 | 14.7 | 14.7 |
|  | ≤ ±4 mmHg | 151 | 85.3 | 85.3 | 100.0 |
| Total |  | 177 | 100.0 | 100.0 |  |

*n*: number.

Supplementary Table 10. Calibration of the prediction rule: Results of linear regression of the observed central venous pressure on predicted central venous pressure

| Coefficient | Estimate | SE | *t* statistic | *P* value | 95% CI Lower | 95% CI Upper |
| --- | --- | --- | --- | --- | --- | --- |
| Intercept | -1.701 | 1.476 | -1.152 | 0.283 | -5.105 | 0.887 |
| Predicted CVP (mmHg) | 1.351 | 0.201 | 6.717 | <.001 | 1.704 | 1.814 |

Dependent Variable: Observed CVP (mmHg).

Model: (Intercept), Predicted CVP (mmHg).

*95% CI*: 95% confidence interval, *CVP*: central venous pressure, *SE*: standard error.

Supplementary Table 11. Recalibration of the prediction rule after the application of a shrinkage factor: Results of linear regression of the observed central venous pressure on predicted central venous pressure

| Coefficient | Estimate | SE | *t* statistic | *P* value | 95% CI Lower | 95% CI Upper |
| --- | --- | --- | --- | --- | --- | --- |
| Intercept | -5.150 | 2.010 | -2.562 | .034 | -9.786 | 1.078 |
| Predicted CVP (mmHg) | 1.652 | 0.249 | 6.632 | <.001 | -0.515 | 2.227 |

Dependent Variable: Observed CVP (mmHg).

Model: (Intercept), Predicted CVP (mmHg).

*95% CI*: 95% confidence interval, *CVP*: central venous pressure, *SE*: standard error.





Supplementary Figure 5. Recalibration plot for the prediction rule after the application of a shrinkage factor the equation slope. Predicted CVP values are grouped into ten deciles. The mean observed CVP (y-axis) is plotted against the mean predicted CVP (x-axis). The dashed line represents the line of identity (y = x), indicating perfect calibration (*i.e.*, ideal 45° line). The dotted line represents the linear regression line (observed vs. predicted). The thick solid line represents the Local Regression Smoothing (LOESS) line. Application of a shrinkage factor resulted in worsened calibration metrics with a slope of 1.678 (95% CI: 1.293 to 2.064, *P* < .001) and intercept of –5.360 (95% CI: –8.471 to –2.249, *P* < .001).
